# Expandable external support device to improve Saphenous Vein Graft Patency after CABG

**DOI:** 10.1186/1749-8090-8-122

**Published:** 2013-05-06

**Authors:** Yanai Ben-Gal, David P Taggart, Mathew R Williams, Eyal Orion, Gideon Uretzky, Rona Shofti, Shmuel Banai, Liad Yosef, Gil Bolotin

**Affiliations:** 1 Tel-Aviv Sourasky Medical Center, Tel-Aviv University, Tel-Aviv, Israel; 2 John Radcliffe Hospital, Oxford University, Oxford, UK; 3 Columbia University Medical Center, New-York, USA; 4 Vascular Graft Solutions LTD, Tel Aviv, Israel; 5 Rambam Medical Center, Haifa, Israel

## Abstract

**Objectives:**

Low patency rates of saphenous vein grafts remain a major predicament in surgical revascularization. We examined a novel expandable external support device designed to mitigate causative factors for early and late graft failure.

**Methods:**

For this study, fourteen adult sheep underwent cardiac revascularization using two vein grafts for each; one to the LAD and the other to the obtuse marginal artery. One graft was supported with the device while the other served as a control. Target vessel was alternated between consecutive cases. The animals underwent immediate and late angiography and were then sacrificed for histopathologic evaluation.

**Results:**

Of the fourteen animals studied, three died peri-operatively (unrelated to device implanted), and ten survived the follow-up period. Among surviving animals, three grafts were thrombosed and one was occluded, all in the control group (p = 0.043). Quantitative angiographic evaluation revealed no difference between groups in immediate level of graft uniformity, with a coefficient-of-variance (CV%) of 7.39 in control versus 5.07 in the supported grafts, p = 0.082. At 12 weeks, there was a significant non-uniformity in the control grafts versus the supported grafts (CV = 22.12 versus 3.01, p < 0.002). In histopathologic evaluation, mean intimal area of the supported grafts was significantly lower than in the control grafts (11.2 mm^^2^ versus 23.1 mm^^2^ p < 0.02).

**Conclusions:**

The expandable SVG external support system was found to be efficacious in reducing SVG’s non-uniform dilatation and neointimal formation in an animal model early after CABG. This novel technology may have the potential to improve SVG patency rates after surgical myocardial revascularization.

## Background

Coronary artery disease (CAD) is the leading cause of death worldwide [1]. The treatment of choice for patients who suffer from severe CAD is coronary artery bypass grafting surgery (CABG) in which the internal mammary arteries (IMAs) and greater saphenous veins are utilized as coronary conduits. While internal mammary arteries carry gratifying long-term patency rates, vein graft failure occurs in approximately 50% five to ten years after surgery with evident atheroma in most of the remaining grafts [2-5]. Although vein graft failure significantly increases patients’ risk of major adverse cardiac events (MACE) [6] and may necessitate coronary re-interventions, the vast majority of all conduits are still saphenous vein grafts (SVG’s).

While early vein graft occlusion is mainly due to technical aspects of the surgical procedure, intermediate and late graft failure results from an irregular remodeling and dilatation of the pressurized thin walled graft [7-9] with subsequent intimal hyperplasia and wall thickening, that reduces the graft’s luminal area and may promote atheroma formation. These developments are thought to be a consequential intrinsic adaptation of the thin walled vein to arterial longitudinal, circumferential and pulsatile flow and pressures [7,10,11].

The concept of external support for vein grafts has been shown to be potentially effective in inhibiting intimal-hyperplasia and wall thickening in several animal studies [12-14]. Unfortunately, due to various technical aspects, preclinical models or study design, this concept has never been assimilated into daily clinical practice. In this report we evaluated a novel expandable external support device, explicitly designed to mitigate causative factors for vein graft failure.

## Methods

Fourteen mature female Assaf sheep weighting 60-80 Kg form the basis of this study.

Surgical procedures were conducted at the Technion, Israel Institute of Technology, Faculty of Medicine, Haifa, Israel after obtaining approval from the institute’s ethical committee for animal experiments. All procedures complied with the Animal Welfare Acts of 1966 (P.L. 89–544), as amended by the Animal Welfare Act of 1970 (P.L. 91–579) and 1976 (P.L. 94–279) and after obtaining approval from the institute’s ethical committee for animal experiments.

The device we evaluated is made from braided cobalt-chromium-nickel-molybdenum-iron alloy fibers, forming an expandable external support apparatus (Fluent, VGS - Vascular Graft Solutions, *Tel Aviv, Israel)*. The aim of the device is to provide vein grafts a shapeable, kink-resistant tight external support, to prevent possible kinking, non-uniform dilation, and wall thickening (i.e. intimal hyperplasia), factors known to cause early and late graft failure.

**Figure 1:**
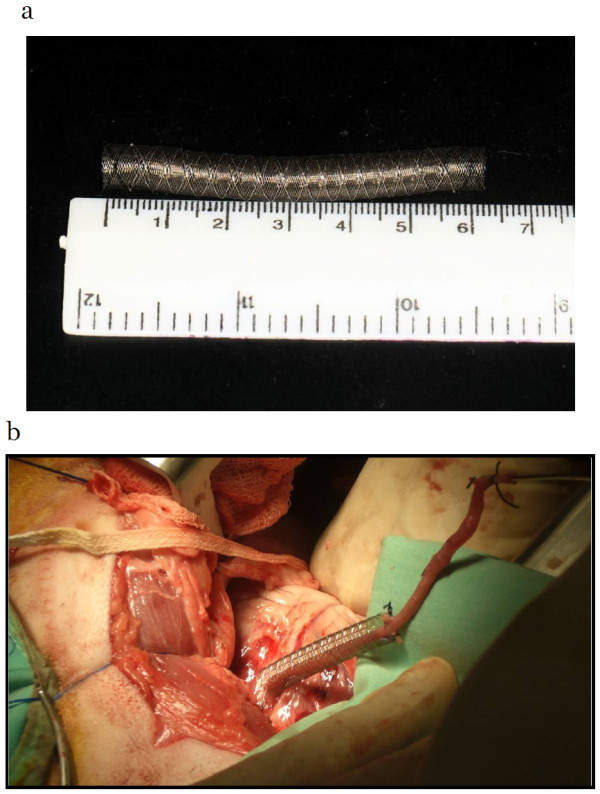
**The compressed external support device. a**: Expandable support device in its compressed configuration. **b**: The device is mounted on the vein graft after first anastomosis.

**Figure 2:**
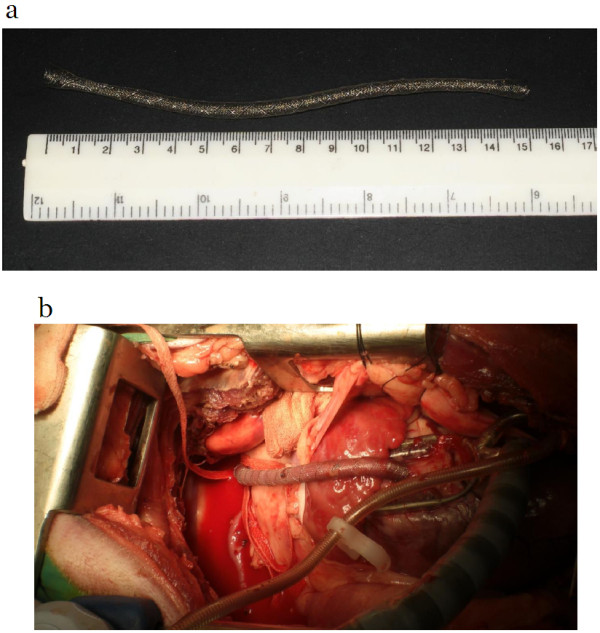
**The stretched external support device. a**: External support device in its stretched configuration; **b**: The device treaded open on the vein graft after the second anastomosis.

Briefly, a compatible device is threaded in a compressed configuration over the vein graft, the device size is determined by measurement of the vein diameter just after harvest, while slightly inflated, and its length is determined just after final trimming of the vein rim, prior to performing the proximal aortic anastomosis (Figure 1). The device is than stretched open manually and shaped by the surgeon to cover the entire vein (Figure 2) while the assistant-surgeon elevates and stabilizes the heart with one hand and the proximal tip of the vein with the other hand. The device’s proximal rim can be easily shortened with surgical Potts scissors for slight differences between device and graft lengths. Final adjustments of the device-graft arrangement are made as the heart returns to its normal volume and position.

All animals were pretreated with buffered aspirin (325 mg/d) and Clopidogrel (150 mg/d) 72 hours prior to surgery and during the follow-up period. Activated Clotting Time (ACT) was measured prior to the performances of the first anastomoses and Heparin 5000 units were administrated to all animals. Additional doses were given to maintain ACT values above 300 seconds, until completion of the surgical procedure. Heparin was not reversed at the end of the procedure.

Animals were placed in dorsal recumbence, and the saphenous vein was harvested through an uninterrupted leg incision, its side branch closed with small size hemoclips. A left lateral thoracotomy at the 3rd or 4th intercostal space was performed with a 20–25 cm long skin Incision with the LAD and larger obtuse-marginal arteries identified following pericardial incision. In each sheep, one vein graft was supported by the device and the other vein graft served as a control, alternating in every procedure, either to LAD or to the left circumflex territories, so that after measuring and trimming both grafts to the desired length, an appropriate device was threaded over only one of the grafts. A stabilizer (Octopus, Medtronic Inc. Minneapolis, MN, USA) was used and grafts were then sutured to the coronary arteries using a continuous 7/0 polypropylene suture. After performing both anastomoses, the proximal coronary arteries were ligated and the device manually stretched open and shaped to cover the entire graft. The pericardium was then loosely reapproximated and the chest was closed with no reversal of heparin. General anesthesia was terminated and the sheep extubated upon resumption of vagal reflexes.

### Angiographic evaluation

Verification of graft patency and quantification of luminal diameter was achieved by immediate post-procedural angiography (t = 0) and at the end of the predetermined follow up (t = 12 weeks post-procedure). Following calibration relative to the 6 Fr diagnostic catheters, all graft angiographic images were divided to seven equal sections, in order to randomly measure the luminal diameters. Percentage coefficient of variance (%CV) was then calculated for each graft to compare diameter variance of the grafts at t = 0 and t = 12 weeks post procedure.

### Histopathologic evaluation

**Figure 3:**
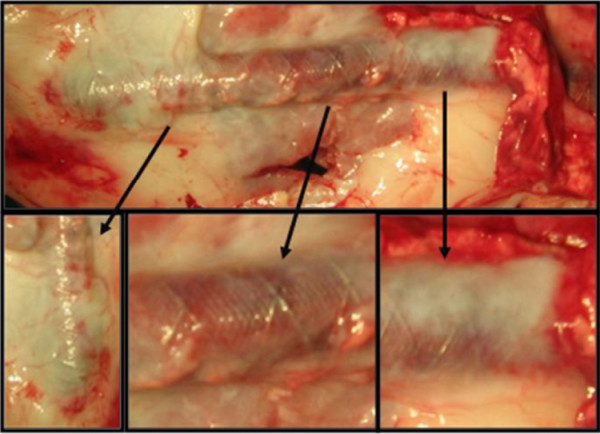
Autopsy documentation 3 months post implantation (Close up sections are indicated with black arrows).

Immediately following the second angiography (t = 12 weeks) animals were sacrificed and grafts were harvested, photographed and examined for inflammation, injury or thrombosis (Table 1 and Figure 3). Grafts were than washed with Dextrose 5% solution and pressure fixated (100 mmhg) in 4% formaldehyde for 72 hours, cut and prepared for standard paraffin (control graft) or plastic embedment and sectioning (grafts with the device) and then stained with H&E or silver staining. Notably, in the first three animals, grafts had smeared layers with partially or fully collapsed wall sections, prohibiting the collection of reliable information. A new pressure fixation method was then implemented on the following seven animals in the study.

**Table 1:** Histopathologic analysis

	**Experimental (supported) grafts**	**Control grafts**
**Inflammation (>1)**	0/10 (0%)	0/10 (0%)
**Injury**	0/10 (0%)	0/10 (0%)
**Thrombosis**	0/10 (0%)	3/10 (30%)

Injury (0–3)

0 = Tunica intima intact.

1 = Tunica intima lacerated, media compressed.

2 = Tunica intima lacerated, media lacerated, adventitia lacerated.

Inflammation (0–3)

0 = no inflammation.

1 = a very mild infiltration of few inflammatory cells (neutrophils/lymphocytes).

2 = moderate infiltration of > 10 inflammatory cells (neutrophils/lymphocytes).

3 = severe infiltration of > 100 inflammatory cells (neutrophils/lymphocytes).

Thrombosis

Organized thrombus in one or more cross section.

Cross- sections were then visualized with a BX51 microscope and thoroughly scanned using the Dotslide *2.1* virtual microscopy system *(Olympus, Germany and Japan).* For each cross section, the lumen, neointima and medial layers were identified and measured with the Dotslide accompanying software. Five to seven cross sections in equal distances from proximal to the distal end were analyzed from each vein, representing 100% of the graft. Of note, plain determination of the average neointimal area along the entire graft from all cross-sections led to falsification of the true angiographic Figure in relation to its possible clinical implications. For example, a case of a distorted irregular graft with severe concentric wall thickening and stenosis proximally, along with significant remodeling and dilatation distally, would be paradoxically interpreted as a “healthy” regular graft. In order to interpret findings according to their true clinical relevance, only the three cross sections with the most extensive neointimal area were compared between supported and control graft.

### Statistical analysis

Due to the nature of the data collection and the desire to demonstrate the effect of external support on the morphology of vein grafts, it was decided to use a measure of variation. Since some correlation between mean values and standard deviations for each graft was evident, it was decided to use the percentage % coefficient of variation (%CV) as the parameter for the analysis. Small %CV values imply that all measurements across a vein are close to each other and thus good uniformity is obtained. Large %CV values indicate large differences in the diameter across the measured vein. This parameter was selected also because either increase (remodeling and dilatation) or decrease (intimal hyperplasia) in vein diameter is possible. Non-parametric matched-pairs (Wilcoxon Sign-Rank) test was used to compare the results from the externally supported and control vein grafts.

## Results

A total of four animals out of the fourteen expired during the follow up period; three deaths occurred peri-operatively and one death occurred three weeks after the procedure. In all four we could not find any device-related event as depicted in Table 2. No device-related complications were noted up until the end of the predefined follow-up period of three months. All together, six sheep received the externally supported vein graft to their LAD territory and a control (non-supported) vein graft to their obtuse marginal (OM) coronary artery, while four animals had an externally supported graft to the OM and a control vein graft to the LAD. All sheep underwent immediate post-operative angiographic evaluation in an attempt to demonstrate the patency and integrity of supported and control grafts.

**Table 2:** Cause of death

**Time of death**	**Cause of death**	**Device related**
Perioperative	Animal expired while elevating the heart before any distal anasthomosis performed or device implantation.	NO
perioperative	Hemodinamyc collapse after post-operative angiography (both grafts patent By angioraphy and PM) suspected narrowing in distal anastomosis of control-graft to LAD.	NO
perioperative	Traumatic intubation and bleeding in the trachea, de-saturation during entire operation (60-80%), multiple VFs prior to the device implantation. Suspected technical error in proximal anastomotic site.	NO
Three weeks after surgery	Sudden death. PM demonstrated viable grafts, intact device and no gross pathologies. Microscopic analysis demonstrated no injury or inflammation to both grafts.	NO

At the end of the three months follow up, a second angiography demonstrated that all ten externally supported grafts were patent compared to nine of the control grafts. On this second angiographic evaluation, we found stenotic lesions, occlusion or irregularities in none of the externally supported grafts versus four of the non supported control grafts, p = 0.043. In late angiography we found no change in device position or in the extent of graft coverage by the device.

**Figure 4:**
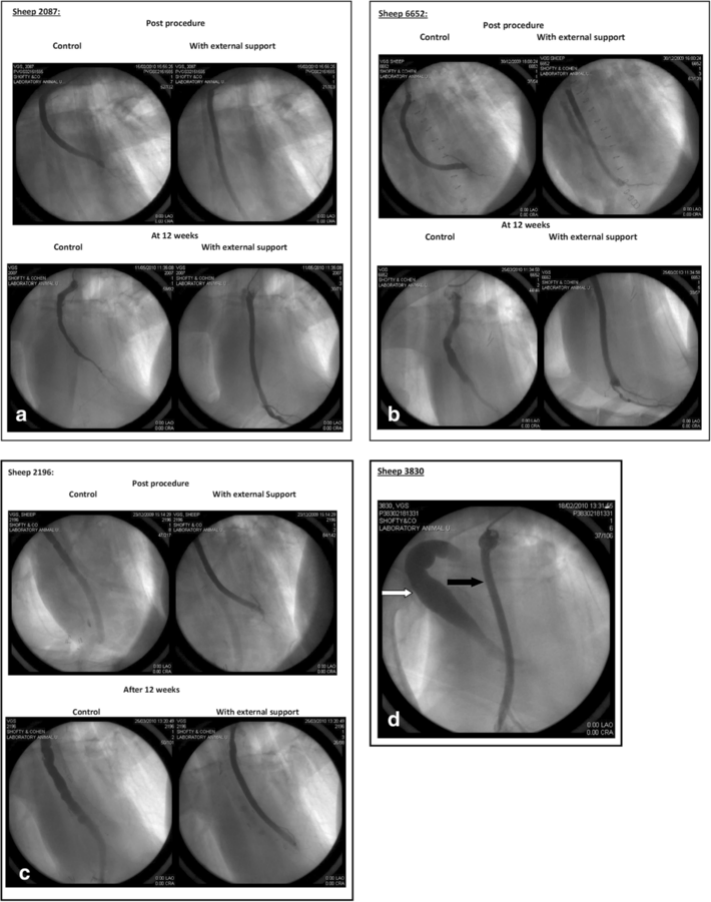
**Early and late post operative angiographic images. a-c**: Angiographic images at t = 0 and at t = 12 weeks for supported and control vein grafts; **d**: Angiographic image of a sheep with aneurysmatic dilatation of the control graft (marked in white arrow) and supported graft (marked in black arrow) after 12 weeks (unfortunately, no angiographic images of the control graft were available for this sheep at t = 0).

Of note, one of the sheep had an aneurysmal dilatation of the control (non-supported) vein at the end of the follow up period, with normal appearance of the supported vein. Regrettably, although both veins appeared quite ordinary during surgery (with photographic documentation) only in this sheep did we fail to demonstrate the unsupported vein during early angiography and it was therefore excluded from angiographic analysis (Figure 4d).

**Figure 5:**
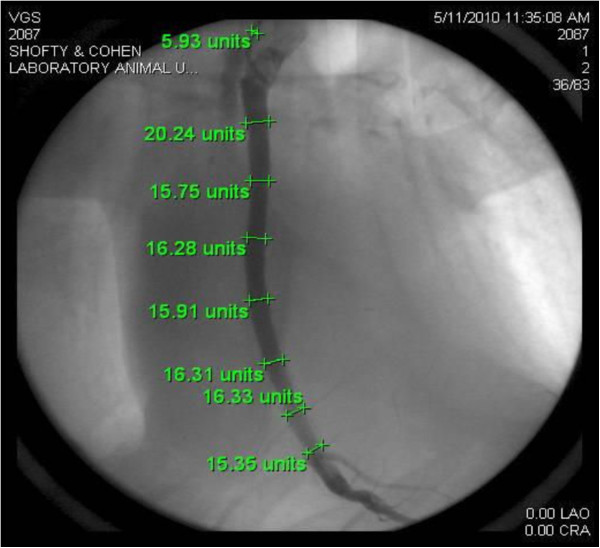
**Angiographic quantification of luminal dimensions in seven sections equally distant from each other.** Variance calculated for each graft at t = 0 and at the end of the follow up (t = 12 weeks).

Quantitative angiographic analysis of early and late angiographic studies was performed (Figure 5) and coefficient of variance (%CV) was then calculated for each graft’s luminal diameters immediately post surgery and at three months of follow up. At t = 0, no significant variance was found between the control and the externally supported grafts (%CV = 7.39 vs. %CV = 5.07, respectively, p = 0.082). Twelve weeks after surgery the %CV in the control grafts increased by 200% (CV% = 22.12) while in the supported grafts CV% decreased by 40% (CV% =3.01, p value < 0.002) representing significant lumen-uniformity differences between the groups. (see Figures 4a- 4d).

In gross pathology and histopathologic evaluation, all grafts and anastomotic sites appeared to be normal, with no apparent difference between grafts in terms of inflammatory cell infiltration and intimal laceration (injury). In the externally supported group the graft’s adventitia was incorporated into the device’s pores, fixated also by a thin layer of lucent connective tissue (Figure 3) with no change of the device position over the graft and without any detected breakage or disintegration points of the device. The angiographic finding of the single occluded control graft was confirmed, while three other control grafts were found with an organized thrombus in one or more cross-sections (Table 1).

**Figure 6:**
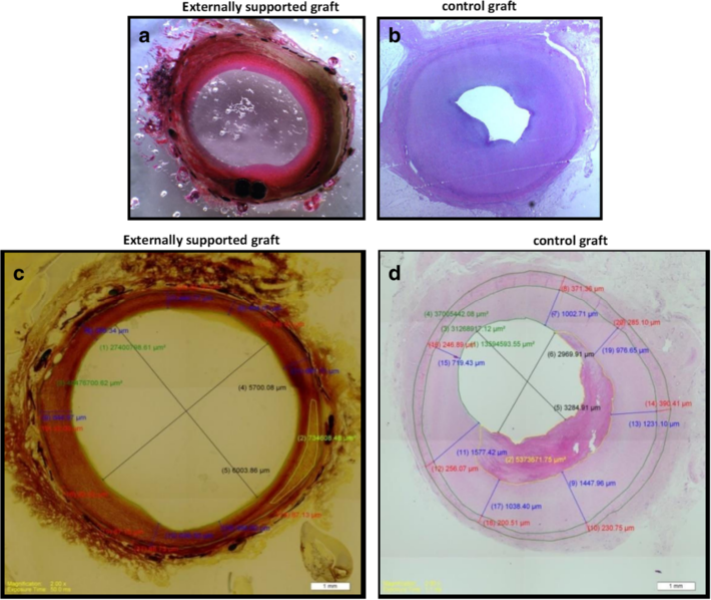
**Histology images at 3 months.** Histologic cross sections at t = 12 weeks of the supported vein grafts (6-**a**, 6-**c**) and control graft (6-**b**, 6-**d**).

Intimal hyperplasia: The measured neointimal area on the histological slide was on average 100% greater in the control grafts compared to the supported grafts (23.056 mm2 vs. 11.196 mm2, p <0.02), with a mean intimal thickness of 0.65 mm in the support group vs. 0.89 mm in the control group, p = 0.11 (Figure 6 illustrating the histologic appearance and analysis of control and externally supported grafts).

Media: The extreme thinness of the media layer and irregular demarcation border with the neointima prevented the extraction of data apart from the assumingly negligible average media thickness of 0.07 mm for the supported grafts vs. 0.25 mm for the control grafts p = 0.007. Lumen: mean luminal-diameter was found to be 5.5 mm in control vs. 4.8 mm in supported grafts p = 0.21 but appropriate analysis of the lumen was difficult, due to the collapsing layers in several histological sections.

To avoid bias of histopathologic results, we calculated all parameters also after excluding the one sheep that had aneurysmal dilatation of the control graft at the end of the follow-up. Intimal area exclusive of the aneurysmal dilated vessel was now 12.5 mm^2^ with the support versus 22.0 mm^2^ in the control vessels, p = 0.04 and intimal thickness of 0.74 mm versus 0.95 mm respectively, p = 0.15.

## Discussion

As vein graft failure occurs in approximately 15-20% during the first post operative year, late patency of SVGs also remains poor at 50% with significant disease in many of the remaining functional grafts [3]. Interfering factors for early patency of SVG’s are kinks from excessive graft length, tortuous pathway and poor surgical and technical dexterity. Other factors are small target vessel size with poor distal runoff, graft-to-coronary diameter mismatch, competitive flow and general co morbidities such as dyslipidemia, hypertension, diabetes mellitus and smoking [3].

Intimal hyperplasia, namely the proliferation of nonfunctional endothelial cells (poor production of nitric oxide, adenosine and other vasomediators) and migration of smooth muscle cells into the vessel wall, is eventually the principal pathophysiological process leading to vein graft failure [15-18]. This process is believed to be the result of the intrinsic adaptation to arterial pressures causing cyclic stretching and increased wall tension of the graft, along with trauma and oxidative stress associated with vein harvest. The diameter mismatch and slow flow in the graft compared to native coronary artery (Hagen-Poiseuill’s law) results in low shear stress inside the vein and promotes the neointimal proliferation [7]. Focal areas of expansion or “remodeling”, further augment wall tension, intimal proliferation and impend laminar flow inside the graft [8,9]. Later on, over-expression of adhesion molecules by the dysfunctional neointima causes monocyte, macrophages and foam cells infiltration and emergence of lipid-laden atherosclerotic plaque formation [2-5,19].

Since first hypothesized by Parsonnet et al. [12] several studies evaluated the possible effect of various external support models such as Dacron [20], polyglactin [21], polyester [14], polytetrafluoroethylene [22], and Nitinol [11] on saphenous vein grafts. These models demonstrated that external reinforcement around saphenous vein grafts may lessen intimal thickening and hyperplasia. Unfortunately, due to various technical or methodological problems none of these external support models was integrated into daily surgical practice.

Additionally, porous external wrapping was shown to promote significant peri-adventitial micro-vasculature, potentially reducing the oxidative stress of the vessel wall [10,21,23], and inversely associated with intimal thickness [13,24]. The degree of constriction was also found to have a possible effect on suppressing intimal hyperplasia [11] and might be related to the symmetry imposed on the vessel wall, enforcing laminar flow inside the graft, reducing the wall tension (Laplace’s law) and the diameter mismatch between the graft and the coronary artery.

In this report we describe the angiographic and histopathological effect of a novel supportive device that was designed to address key promoting factors for early and intermediate vein graft failure: kink, torsion, asymmetric dilatation-remodeling and the formation of thick ineffectual neointima. This device is tangentially elastic and un-kinkable but shapeable in its long axis to control pathway and position of the graft. The device is deployed in a slightly compressive manner, with no mounting appliances or adhesive materials.

With no immediate angiographic differences, after three months the externally supported grafts maintained their angiographic appearance and luminal regularity (compared to initial angiography), while the control grafts had an irregular appearance, with a significant change in the luminal coefficient of variance. Three non supported grafts had evident stenotic lesions (one graft was occluded). In histopathologic analysis, no difference between supported and control grafts was found in terms of vessel wall trauma or inflammation; yet, supported grafts had a significantly smaller (approximately 50%) intimal area. Furthermore there was evident thrombus formation within four of the controls versus none in of the supported group.

This report has evident limitations with its sample size and follow up period, but these are no different than the other preclinical studies performed to evaluate the role of external support on vein grafts. Several years of follow up may be needed to fully demonstrate the exact impact of external support on vein graft occlusive disease. However, with the well defined information on the pathological phases that cause vein graft failure, adequate assumptions may well be made on final stages (atheromatous plaque), based on attenuation of earlier (dilation-remodeling and intimal hyperplasia) occurrences, such as the differences we found after three months of follow up. In addition, the majority of the surviving animals that entered the angiographic and histopathologic evaluation had the device applied to the grafts anastomosed to the LAD (six out of ten sheep), hence a potential difference may have theoretically contributed to the different outcome between supported and non supported grafts due to possible dissimilarity in flow characteristics between different coronary territories. Importantly, some covariates (mainly technical), such as coronary target diameter, angiographic appearance and vascular bed, were not included in the analysis. These technical parameters might affect progression of occlusive disease in the both externally supported and control grafts. Finally, this study’s animal (sheep) model or the abrupt occlusion (ligation) that was inflicted on native coronary targets may not accurately reflect a human heart with chronic atherosclerotic disease. Still, among recent studies on venous external support, to our knowledge only in the current study was an actual CABG model actually used.

Despite the unprecedented success of CABG procedures in the treatment of CAD, the main mitigating factor against excellent long-term results remains poor vein-graft patency rates. Vein-graft failure is a major cause for repeated surgical procedures, PCI’s, hospitalizations and other adverse events. However even with the growing awareness and dedication of the surgical community to using multiple arterial grafts, saphenous veins are, and will continue to be in the foreseeable future, the predominant grafts employed during surgical revascularization.

## Conclusions

Our current findings suggest that the expandable external support device that was examined in a realistic sheep model of surgical myocardial revascularization proved efficacious in reducing vein graft irregularity and intimal hyperplasia and may have the potential to improve patency and longevity of vein grafts deployed during clinical coronary revascularization. In a further clinical investigation, the first trial with this expandable support system is currently being conducted in the UK, to determine the potential of this device in future surgical practice.

## Abbreviations

CABG: Coronary artery bypass grafting; LAD: Left anterior descending; LCX: Left circumflex; OM: Obtuse marginal; CV: Coefficient-of-variance; SVG: Saphenous vein graft; MACE: Major adverse cardiac events; ACT: Activated clotting time; CAD: Coronary artery disease; PCI: Percutanous coronary intervention.

## Competing interests

Yanai Ben-Gal MD - Medical director and ownership interest in VGS.

David P. Taggart PHD, FRCS - Scientific advisory board of VGS, has ownership interest in VGS.

Mathew R. Williams MD - Scientific advisory board of VGS, has ownership interest in VGS.

Eyal Orion MD – CEO of VGS, investor and has ownership interest in VGS.

Shmuel Banai MD - Scientific advisory board of VGS, has ownership interest in VGS.

Rona Shofti PHD- Has ownership interest in VGS.

Gideon Uretzky MD -None.

Lyad Yosef BSc - Has ownership interest and employee of VGS.

Gil Bolotin MD, PHD - Scientific advisory board of VGS and has ownership interest in VGS.

## Authors’ contributions

YBG conceived of the study, design, writing the manuscript and revising. DT, helped in Conception, design and writing. MW helped to draft the manuscript. EO conceived of the study, design, analysis of data, active participating in the study and helped to draft the manuscript. GU and helped to draft the manuscript, RS active participation in the study SB helped to draft the manuscript. LY active participation in the study, analysis of data. GB active participation in the study, helped in conception, design and writing. All authors read and approved the final manuscript.
